# Ultrasonic Vocalizations in Adult C57BL/6J Mice: The Role of Sex Differences and Repeated Testing

**DOI:** 10.3389/fnbeh.2022.883353

**Published:** 2022-07-14

**Authors:** Marika Premoli, Valeria Petroni, Ronald Bulthuis, Sara Anna Bonini, Susanna Pietropaolo

**Affiliations:** ^1^Department of Molecular and Translational Medicine, University of Brescia, Brescia, Italy; ^2^Univ. Bordeaux, CNRS, INCIA, UMR 5287, Bordeaux, France; ^3^Metris B.V., Hoofddorp, Netherlands

**Keywords:** ultrasonic communication, social behaviors, sex, repeated testing, isolation

## Abstract

Ultrasonic vocalizations (USVs) are a major tool for assessing social communication in laboratory mice during their entire lifespan. At adulthood, male mice preferentially emit USVs toward a female conspecific, while females mostly produce ultrasonic calls when facing an adult intruder of the same sex. Recent studies have developed several sophisticated tools to analyze adult mouse USVs, especially in males, because of the increasing relevance of adult communication for behavioral phenotyping of mouse models of autism spectrum disorder (ASD). Little attention has been instead devoted to adult female USVs and impact of sex differences on the quantitative and qualitative characteristics of mouse USVs. Most of the studies have also focused on a single testing session, often without concomitant assessment of other social behaviors (e.g., sniffing), so little is still known about the link between USVs and other aspects of social interaction and their stability/variations across multiple encounters. Here, we evaluated the USVs emitted by adult male and female mice during 3 repeated encounters with an unfamiliar female, with equal or different pre-testing isolation periods between sexes. We demonstrated clear sex differences in several USVs' characteristics and other social behaviors, and these were mostly stable across the encounters and independent of pre-testing isolation. The estrous cycle of the tested females exerted quantitative effects on their vocal and non-vocal behaviors, although it did not affect the qualitative composition of ultrasonic calls. Our findings obtained in B6 mice, i.e., the strain most widely used for engineering of transgenic mouse lines, contribute to provide new guidelines for assessing ultrasonic communication in male and female adult mice.

## Introduction

Mice emit ultrasonic vocalizations (USVs) to communicate with each other in a social context during defined phases of their life: in newborn (i.e., during the first 15 post-natal days) to summon the mother, in the juvenile phase (i.e., between 3 and 7 weeks of age) during playing in same-sex dyads and at adulthood most commonly during male-female or female-female interactions (Lahvis et al., 2011; Arriaga and Jarvis, 2013; Caruso et al., 2020). USVs have a frequency range between 30 and 110 kHz and are of innate nature, since it has been demonstrated that mice are not vocal learners (Kikusui et al., 2011; Mahrt et al., 2013). At all ages, different types of vocalizations exist with specific spectrographic characteristics that several researchers have struggled to classify using different technologies and approaches (Holy and Guo, 2005; Gaub et al., 2016; Grimsley et al., 2016; Premoli et al., 2021a). However, the understanding of the precise significance of different types of calls is still unknown to date, and additional data are needed to better unravel this issue, allowing an essential step forward in the field of mouse behavioral neuroscience.

Studies on USVs have become a widely used behavioral assay to monitor the emotional state of mice (Simola and Granon, 2019) and their sociability. The research interest in USVs is indeed justified from both ethological and preclinical points of view: a growing number of studies have applied USVs as a valuable tool to study pathologies characterized by deficits in communication and sociability in mouse models (such as autism spectrum disorder, ASD) and to investigate the effects of therapeutic approaches, since USVs can be modulated by diverse pharmacological treatments (Premoli et al., 2021b). A large part of studies on adult ultrasonic communication have focused on USVs emitted by male mice, since these are more commonly employed than those by females for behavioral phenotyping of animal models of ASD and avoid the well-known impact of the estrous cycle on females' USVs (Moles et al., 2007). USVs produced by adult mice during male-female dyadic interactions are also the most extensively characterized, and they allow for easier identification of the emitting animal, since it has been demonstrated that in this context USVs are mostly produced by male mice to attract female mice (Sugimoto et al., 2011; Hammerschmidt et al., 2012; Egnor and Seagraves, 2016).

Nonetheless, USVs can also be emitted by adult females: recent studies have described that adult receptive female mice produced USVs either in the presence of male urines enriched with pheromones or in groups of four mixed-sex individuals (Neunuebel et al., 2015; Demir et al., 2020). However, in these studies, the female mice were assessed under experimental conditions maximizing the expression of their sexual interest, including being tested in a receptive estrous state, during the dark phase, and for long sessions (more than 30 min). Even under these conditions, the largest proportion of USVs registered during group interactions was produced by males (Neunuebel et al., 2015). Instead, female mice show their most prominent vocalizing abilities during adult female-female dyadic interactions, and in these cases USVs are used to establish affiliative relationships (Moles and D'Amato, 2000; Moles et al., 2007; Zala et al., 2017). In particular, Moles and D'Amato (2000); Moles et al. (2007) studied USVs of adult female mice in a resident-intruder setting, i.e., the resident being isolated in the testing cage 3 days before assessing the USVs with a female intruder. They demonstrated that in these experimental settings most of the USVs are uttered by the resident and suggested that the calls can facilitate proximity with the intruder and reduce its potential aggressiveness. Female USVs in this context can be also used as an index of sociability and social memory, since (i) a strong positive correlation was found between the number of calls and the time spent by the resident female mouse sniffing the intruder, and (ii) a marked decline was observed in the number of USVs emitted by a resident female mouse when exposed multiple consecutive times to the same female intruder. USVs are mostly emitted during close contacts and approach behaviors in female-female interactions and male-female encounters (Ferhat et al., 2015, 2016). Nonetheless, the precise link between multiple USV characteristics and other social behaviors is still not fully understood in adult female mice, as in male mice.

Several studies have tried to analyze differences in USVs between male and female mice (Hammerschmidt et al., 2012; von Merten et al., 2014; Zala et al., 2017; Matsumoto and Okanoya, 2018; de Chaumont et al., 2021), also with novel technical approaches such as deep learning networks (Ivanenko et al., 2020), yielding to the emergence of a variety of either quantitative or qualitative differences (or both) without, so far, a univocal pattern. Divergences among these studies mainly arise from differences in testing procedures, e.g., sex of the stimulus animal and pre-testing isolation conditions. The sex of the “receiver” is known to critically modulate several characteristics of USVs of the “emitter” in mice of both sexes (Zala et al., 2017); for instance, quantitative sex differences in USVs were described between female-female and male-female interactions (von Merten et al., 2014), while both quantitative and qualitative differences were observed in same-sex interactions (de Chaumont et al., 2021). Also, most of the studies on female USVs have applied relatively long periods (more than 24 h) of pre-testing isolation in order to induce a resident status in the subject and assure the identification of the emitter (Moles et al., 2007; Hammerschmidt et al., 2012). In contrast, pre-testing isolation is not commonly applied in USV studies on adult male mice, as this manipulation is not necessary to induce their USV production. In general, isolation is known to alter USV emission in adult mice (Lefebvre et al., 2020; Zhao et al., 2021) and to modulate the correlation between USVs and other social behaviors (Chabout et al., 2012). Finally, most of the studies on sex differences in USVs have employed single testing sessions or multiple repeated sessions but with the same social stimulus in order to assess habituation (Moles et al., 2007). Hence, it is not clear whether sex differences in ultrasonic communication may be dependent on testing experience or are a stable trait across multiple social encounters with an unfamiliar stimulus.

Here, we performed an extensive quantitative and qualitative characterization of sex differences in USVs emitted by adult C57BL/6J (B6) mice. To this end, we compared the USVs uttered either by an adult male or female toward the same type of stimulus, i.e., an adult CD1 female. The female mice were isolated for 3 days before testing in order to acquire the status of resident, i.e., becoming the major emitter of USVs during interaction with a female intruder. Their USVs were compared with those of males that were either isolated for the same duration before testing (study 1) or only habituated to the testing cage for 10 min before tests (study 2). These two studies allow us to evaluate sex differences respectively (1) in the same resident-intruder settings, thus controlling for isolation effects and (2) using the most common (and practically more suitable) experimental settings for USV assessment used in previous studies with ASD mouse models, i.e., in the resident-intruder context for females and without pre-testing long isolation in males (Pietropaolo et al., 2011, 2014; Hebert et al., 2014; Oddi et al., 2015; Gaudissard et al., 2017; Gauducheau et al., 2017; Fyke et al., 2021). In both studies, three subsequent social tests with a novel intruder were performed with an interval of 7–10 days in order to evaluate the potential stability of sex differences and their dependency on previous testing experience without confounding effects of social memory. For each encounter, social affiliative behaviors were also assessed in order to evaluate their potential link with USV changes. Estrous cycle phases were assessed for experimental female subjects and female stimuli before each social encounter to control for potential hormonal modulation of social interaction and communication (Moles et al., 2007; Hanson and Hurley, 2012; Egnor and Seagraves, 2016; Kim et al., 2016).

We chose B6 mice as the experimental subjects of our study because of the well-known relevance of this strain for behavioral neuroscience due to its large use for engineering genetically modified mouse lines. Stimulus females for all social tests were instead chosen from the CD1 strain because of its common use in social studies (Moles and D'Amato, 2000; Moles et al., 2007), especially those using genetic mouse models of ASD (Hebert et al., 2014; Pietropaolo et al., 2014; Oddi et al., 2015; Gaudissard et al., 2017; Gauducheau et al., 2017; Lemaire-Mayo et al., 2017; Fyke et al., 2021). This strain is preferentially employed in social interaction tests, since it is characterized by high levels of sociability and it facilitates behavioral analysis during social encounters with B6 animals because of its albino phenotype.

## Materials and Methods

### Animals and Housing Conditions

Forty adult male and female C57BL/6J mice (10–12 weeks old, *n* = 10 per sex in each experiment), used as experimental subjects, and forty adult CD1 females, used as social stimuli, were purchased from Janvier (Le Genest St Isle, France). Upon arrival at our animal facility at Bordeaux University they were all housed in same-sex and same–strain groups of 5 individuals in standard polycarbonate cages (37 × 21 × 15 cm in size; Tecniplast, Limonest, France) and provided with sawdust bedding (SAFE, Augy, France) enriched with cotton nestlets. Food chow (SAFE, Augy, France) and water were provided *ad libitum*. The animals were maintained in a temperature- (22°C) and humidity- (55%) controlled vivarium under a 12:12 h light–dark cycle (lights on at 7 a.m.). The mice were left undisturbed for 2 weeks upon their arrival before the behavioral tests began.

As illustrated in Supplementary Figure 1, two separate groups of mice were used for the two experiments of the study, each consisting of 20 B6 experimental subjects (10 male mice and 10 female mice) and 20 CD1 stimulus females. The CD1 female mice were all naïve to social experience with B6 mice at the time of the first testing session; each female stimulus was employed for a total of 3 times for each experiment but only once for each testing session. B6 mice of either sex encountered a novel female at each session. Separate batches of CD1 female mice were employed for male-female and female-female interactions in each experiment, so that a CD1 stimulus encountered either B6 female or male mice during the 3 sessions. In experiment 1, both male and female B6 subjects were single-caged for the same time (72 h) in the test cage before each testing session to assess social behaviors and USVs: this allowed for us to assess sex differences in the same resident-intruder settings and pre-testing social isolation conditions. In experiment 2, the male mice were subjected to 10 min of isolation in the test cage, and their social behavior was compared with that of female mice exposed to 72-h pretesting isolation: this comparison served to evaluate sex differences under conditions that are commonly employed to assess male and female USVs in ASD mouse studies (Hebert et al., 2014; Pietropaolo et al., 2014; Oddi et al., 2015; Gaudissard et al., 2017; Gauducheau et al., 2017; Lemaire-Mayo et al., 2017; Fyke et al., 2021) and that are also more suitable for male behavioral assessment. USVs can in fact be also evaluated in male-female interactions without inducing a resident state in the male [e.g., Hebert et al., 2014; Oddi et al., 2015; Gaudissard et al., 2017; Lemaire-Mayo et al., 2017; Fyke et al., 2021], thus avoiding applying a social isolation period of at least 72 h that could interfere with several other behaviors. In contrast, female mouse USVs are most commonly assessed in a resident-intruder setting, at least in female-female dyadic interactions. In both experiments, after each testing session, the experimental mice were re-housed in groups with the same cagemates. The CD1 stimulus mice were kept under grouped conditions during the entire duration of the study.

### Behavioral Procedures

Behavioral testing was carried out during the light phase of the cycle. All the experimental procedures were performed in accordance with the European Communities Council Directive 2010/63/EEC and approved by the Local Ethical Committee (“Comité d'Ethique pour l'experimentation animale de Bordeaux”, CE 50) and the French Ministry (“Ministere de l'enseignement superieur de la recherché et de l'innovation”).

Social behavior and ultrasonic communication were assessed in a 33 × 15 × 14 cm plastic cage with 3 cm of sawdust and a metal flat cover during 3 testing sessions of 3 min each and with an interval of 7–10 days. In experiments 1 and 2, the female B6 subjects were isolated in the testing cage for 72 h prior to testing in order to induce the status of resident in the adult female mice and therefore promote the emission of ultrasonic vocalizations (USVs) toward an adult female intruder (Moles et al., 2007). The male B6 subjects were isolated either for 72 h (experiment 1) or for 10 min (experiment 2) in the testing cage before each social encounter. In all the experiments, an unfamiliar adult female CD1 stimulus was then introduced into the testing cage of either male or female subjects and left there for 3 min. Previous studies alternately anesthetizing each pair member have shown that in these experimental settings adult stimulus females do not emit ultrasonic vocalizations (USVs) that are instead mostly uttered by the experimental male (Whitney et al., 1973; Maggio and Whitney, 1985) or the resident female (Maggio and Whitney, 1985; D'Amato and Moles, 2001). The lack of concomitant emission of USVs by the two interacting animals was indeed confirmed here by additional inspection of spectrograms, excluding the presence of “double calls”, i.e., overlapping in their timing, but with different, non-harmonic characteristics (e.g., different peak and mean frequency, modulation). After each testing session of both experiments, the experimental and stimulus mice were returned to their home cages and kept with their original cagemates until the subsequent testing session.

The testing sessions were recorded with a camera placed on the side of the cage, and videos were analyzed with Observer XT (Noldus, The Netherlands). One observer who was unaware of the sex and experimental assignment of the animals scored the behavior of the test B6 mice only, quantifying the time spent performing the following behaviors (Pietropaolo et al., 2011, 2014; Oddi et al., 2015; Gaudissard et al., 2017; Gauducheau et al., 2017):

- affiliative behaviors: nose/anogenital/body sniffing (sniffing the head and snout of the partner/its anogenital region/any other part of the body), contact with the partner (traversing the partner's body by crawling over/under from one side to the other or allogrooming)
- nonsocial activities: rearing (standing on the hind limbs and sometimes with the forelimbs against the walls of the cage), exploring the cage (locomotion and wall rearing), digging, grid-climbing, self-grooming (the animal licks and mouths its own fur).

An ultrasonic microphone, UltraSoundGate Condenser Microphone CM 16 (Avisoft Bioacoustics, Berlin, Germany), was mounted 2 cm above the cover of the testing cage; it was connected *via* an UltraSoundGate 116 USB audio device (Avisoft Bioacoustics) to a personal computer, with which acoustic data were recorded with a sampling rate of 250 kHz in 16-bit format with Avisoft Recorder (version 2.97; Avisoft Bioacoustics). The recordings were then transferred to Avisoft SASLab Pro (Version 5.20; Avisoft, Berlin, Germany) and Fast Fourier transformation was applied (512 FFT length, 100% frame, Hamming window, and 75% time window overlap).

**Figure 1:**
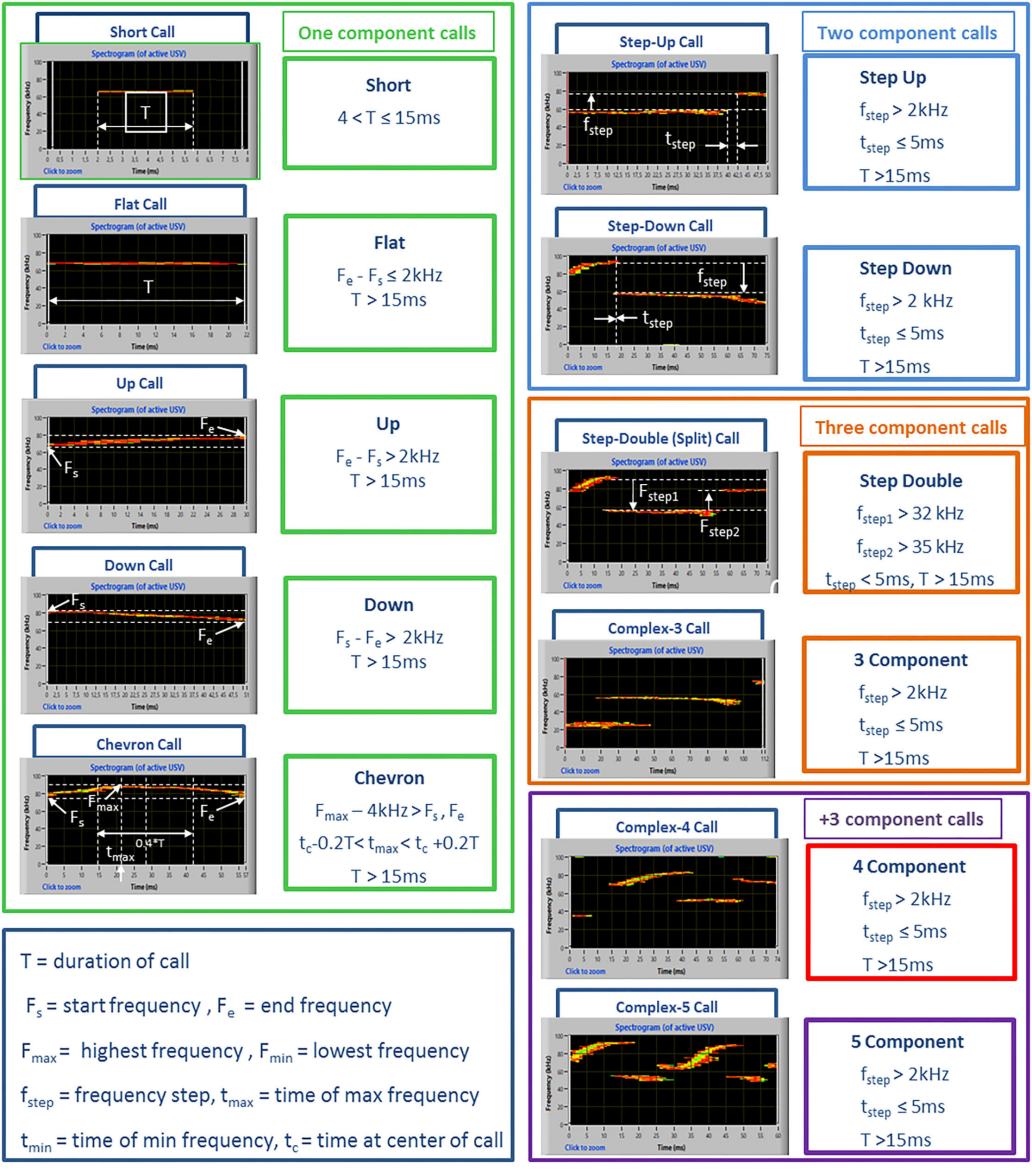
Examples of ultrasonic call types used to classify USVs in the study. The call types were automatically classified using the software Sonotrack and based on the parameters described above. Definitions of the call types were mutually exclusive. Overlap of components was removed when more than 70 % to prevent wrong call durations. Short gaps between components in both frequency (≤6 kHz) and time (≤ms) were interpolated (gaps can be caused by changes in microphone sensitivity or direction of vocalization). Complex “3 component” and “+3 component” calls were summed up into a “complex tot” category.

Spectrograms were generated with Avisoft at a frequency resolution of 488 Hz and a time resolution of 0.512 ms. Signals below 30 kHz were cut to reduce background noise to 0 dB (Premoli et al., 2019). For USV detection, an interactive function with section labels was used. This tool permits to define manually USV borders by inserting section labels, and it is useful when automatic threshold-based USV separation may not work satisfactorily because of ambient noise or because of poorly structured vocalizations (manual guide of Avisoft Bioacoustics). Number, mean duration, peak frequency, and peak amplitude were calculated for each vocalization together with the calling time of the mice based on previous studies (Wohr et al., 2011). Call subtypes were also determined for a more detailed qualitative analysis. For this purpose, USVs were automatically classified with the Sonotrack Call Classification (version 1.4.7, Metris B.V., The Netherlands) software, using the categories described in detail in Figure 1, based on previous literature on mouse USVs (Caruso et al., 2020). To deal with background noise and artifacts in the ultrasound recordings, the Sonotrack Call Classification software applies various filters to remove unwanted signals such as white noise and artificial sound sources. In addition, a logic filter is used that further processes the recorded signal by removing sounds that are too short or appear at many frequencies at the same time. The logic filter also reduces echo that is found in the recording and merges spectral elements that are interrupted by a very short time and a frequency gap.

On each testing day, the vaginal estrous phases of both testing and stimulus female mice were assessed from the analysis of their vaginal smears (Caligioni, 2009). In both experiments, all stimulus females used for male-female interactions were in non-receptive diestrous phase in order to minimize mounting attempts and other sexual behaviors that could confound the evaluation of sex differences in USVs and social behaviors. The stimulus females for female-female interactions were either in diestrous (non-receptive) or estrous (receptive) phase, and their assignment was counterbalanced depending on the estrous phase of the experimental subjects (i.e., approximately half of the intruders in the estrous phase encountered a resident in estrous and the other half was assigned to a resident in diestrous; the same design was applied to the intruders in diestrous). The estrous phases of the female residents and intruders for each testing session of female-female interactions are illustrated in Table 1.

**Table 1:** Estrous phase of the resident B6 female mice and the CD1 stimulus female intruders for each testing session.

**Experiment**	**Estrous phase**	**Session 1**		**Session 2**		**Session 3**	
		**Estrous**	**Diestrous**	**Estrous**	**Diestrous**	**Estrous**	**Diestrous**
1	Resident	5^*^	5	4^*^	6	4^*^	6
1	Intruder	5	5^*^	4^*^	6	5^*^	5
2	Resident	4	6	7	3	7	3
2	Intruder	5	5	6	4	5	5

*While the female CD1 intruders used for assessing males' USVs and social behaviors in male-female interactions were all in diestrous phases, the intruders used for female-female interactions were either in diestrous or estrous phase. Their assignment on each testing day was counterbalanced according to the estrous phase of the B6 experimental female mice (i.e., approximately half of the intruders in estrous phase encountered a resident in estrous and the other half was assigned to a resident in diestrous; the same design was applied to the intruders in diestrous). The female CD1 stimuli at the time of the first testing session were naïve to encounters with mice of different strains (i.e., they have had pre-testing social interactions only with their same-sex and same–strain cagemates); they were used for a total of 3 times on the 3 subsequent testing sessions, but they were tested only once for each session. Separate batches of stimulus females were used for female-female interactions and male-female tests, and for each experiment (for a total of 40 CD1 female and 10 B6 male + 10 B6 female mice for each experiment)*.

* *One female B6 was excluded from the analysis of social behaviors because she was a statistical outlier on the time spent in affiliative behaviors (based on Grubbs' ESD test)*.

### Statistical Analyses

Normality of data distribution was confirmed by Shapiro-Wilks test for each sex and testing session and for each variable of interest. Behavioral data from each experiment were separately analyzed by ANOVA with sex as the between-subject factor and testing session as the within-subject factor. Furthermore, behavioral data from the female mice in each testing session were subjected to an additional ANOVA with the estrous phase (estrous or diestrous) of the experimental B6 female and the estrous phase (e.g., Supplementary Figures 2, 3) of the stimulus CD1 female as the between subject-factors. The analysis of the data from male mice did not include the estrous phase of the stimulus, since all CD1 females selected for testing males were in diestrous phase. A further ANOVA with experiment as the additional between-subject factor was performed on the data from female mice only in order to quantify the replicability of the female phenotype (under the same testing conditions) across the two experiments.

*Post-hoc* comparisons (Fisher's LSD test) and separate ANOVAs were performed when appropriate. All the analyses were conducted using the software Statview and SPSS, and α was set at 0.05. The data were inspected for exclusion of outliers (by Grubbs' ESD test adapted for small sample size). Outlier values were excluded only from a specific dataset (e.g., body sniffing time on session 1), except for the analysis of repeated measures, when values for all the 3 sessions had to be excluded for the affected variable. The results are expressed as mean ± SEM throughout the text. Individual data of social behaviors and USV parameters are also provided for all the animals in Supplementary Figures 4, 5; in addition, the individual composition of call types is illustrated in Supplementary Figures 6, 7 for half (i.e., 5 over 10) of individuals for each sex emitting the higher rates of USVs in each experiment.

## Results

### Experiment 1: Same Pre-Testing Isolation Time in Both Sexes

#### Sex Differences: Social Interaction and USVs

**Figure 2:**
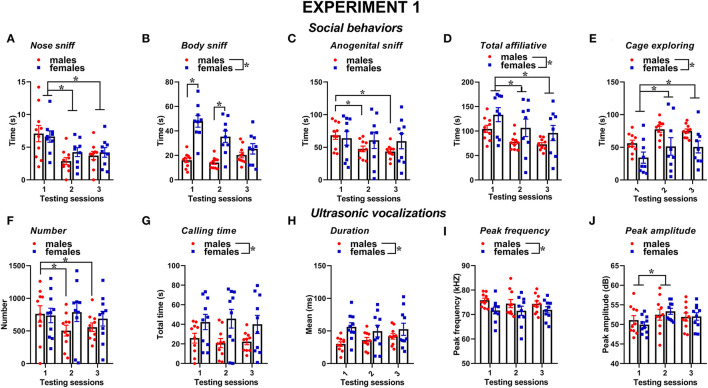
Sex differences in social behaviors and USVs in experiment 1 (same pre-testing isolation). All behaviors were scored in the male and female experimental B6 subjects **(A–E)** toward a female CD1 stimulus during 3 testing sessions of 3 min each (inter-session interval: 7–10 days). Time spent performing social behaviors and cage exploration was scored from video files by an observer who was unaware of the sex of the subjects and their experimental assignment. Affiliative behaviors refer to all sniffing + crawl under/over + allogrooming (refer also to definitions in the text). Cage exploration refers to locomotion in the testing cage and wall rearing. USVs were quantified by spectrographic analysis using the software Avisoft SASLab Pro **(F–J)**. They were emitted either by male or female experimental B6 subjects toward a female CD1 stimulus during the 3 testing sessions of 3 min each. Data are mean ± SEM. *N* = 10 before exclusion of statistical outliers by Grubb's test for small samples.*p < 0.05. * Refers to a nonsignificant tendency (0.05 < *p* ≤ 0.09). Sex differences are reported as * in each graph legend when a significant main effect of sex was detected in the absence of any interaction with testing session.

Social behaviors were overall more markedly expressed in the female mice than in the male mice and tended to decrease with testing sessions; furthermore, sex differences depended on specific type of considered social behavior (Figures 2A–E). The time spent performing nose sniffing was overall similar in mice of both sexes and tended to decrease with repeated testing sessions [sex effect and its interaction, n.s., session effect: *F*_(2, 34)_ = 10.18, *p* < 0.001, Figure 2A]. The female mice displayed more body sniffing than the male mice [sex effect: *F*_(1, 17)_ = 22.31, *p* < 0.001, Figure 2B], and this effect was mostly observed during the first 2 sessions, since on the 3rd encounter body sniffing decreased in the female mice but increased in the male mice [interaction sex × session: *F*_(2, 34)_ = 18.59, *p* < 0.0001, Figure 2B; *post-hoc*: *p* < 0.05]. The time spent performing anogenital sniffing did not differ overall between sexes, but it decreased with testing sessions in the male mice only [overall interaction sex × session: *F*_(2, 34)_ = 2.78, *p* < 0.07, session effect on the male mice: *F*_(2, 18)_ = 14.68, *p* < 0.01; on the female mice: ns, Figure 2C]. The female mice showed a tendency to display more affiliative behaviors than the male mice and explored significantly less the testing cage than males [sex effect, respectively: *F*_(1, 17)_ = 3.22, 6.43, *p* = 0.09 and *p* < 0.05; Figures 2D,E]. In mice of both sexes, the levels of affiliative behaviors tended to decrease with testing sessions while those of cage exploration increased [session effect, respectively: *F*_(2, 34)_ = 25.01 and 10.26, *p* < 0.001; Figures 2D,E].

Several characteristics of USVs differed between the two sexes (Figures 2F–J). Although the number of USVs emitted was not significantly different (Figure 2F), the total calling time and the mean duration were higher in the female mice [sex effect, respectively: *F*_(1, 18)_ = 4.85 and 4.44, *p* < 0.05; Figures 2G,H], while the peak frequency tended to be lower than in the male mice [sex effect: *F*_(1, 18)_ = 3.62, *p* = 0.07; Figure 2I]. All the USV parameters did not significantly change across the testing sessions, with the exception of peak amplitude that increased from the first to the second session in mice of both sexes [session effect: *F*_(2, 36)_ = 4.27, *p* < 0.05; Figure 2J]. Although the interaction sex × session did not reach statistical significance, it was evident that the number of USVs decreased across the testing sessions in the male mice only [separate ANOVAs on the male mice: *F*_(2, 18)_ = 9.59, *p* < 0.01; in the female mice:, ns; Figure 2F].

**Figure 3:**
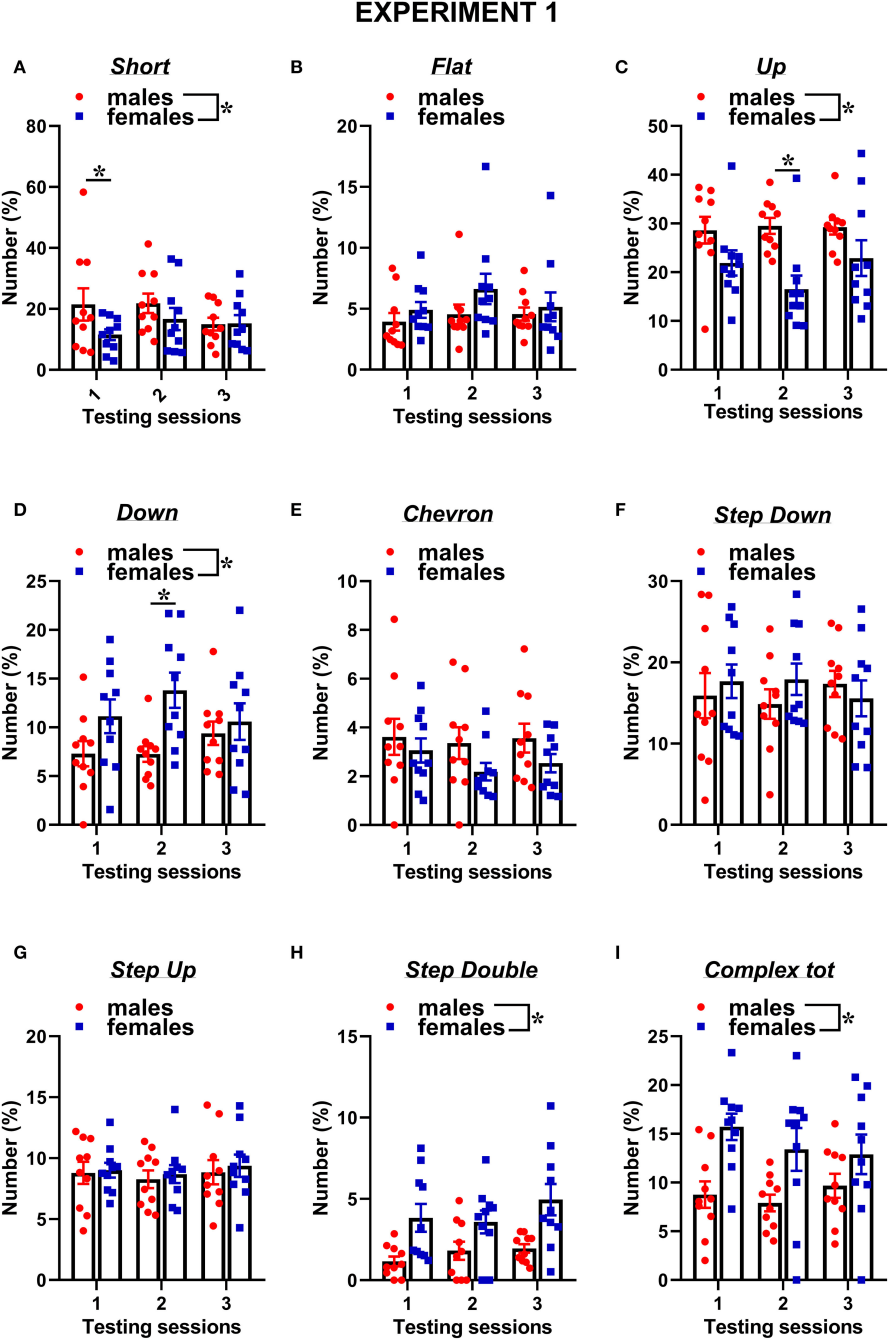
Sex differences in ultrasonic call types in experiment 1 (same pre-testing isolation). **(A–I)** The different call types were automatically classified as detailed in Figure 1. Complex tot = complex 3 + complex 4+ complex 5. Data are expressed as percentages over the total number of USVs for each sex and session. Data are mean ± SEM. *N* = 10 for each sex. *p < 0.05. * Refers to a nonsignificant tendency (0.05 < *p* ≤ 0.09). Sex differences are reported as * in each graph legend when a significant main effect of sex was detected in the absence of any interaction with testing session.

**Figure 4:**
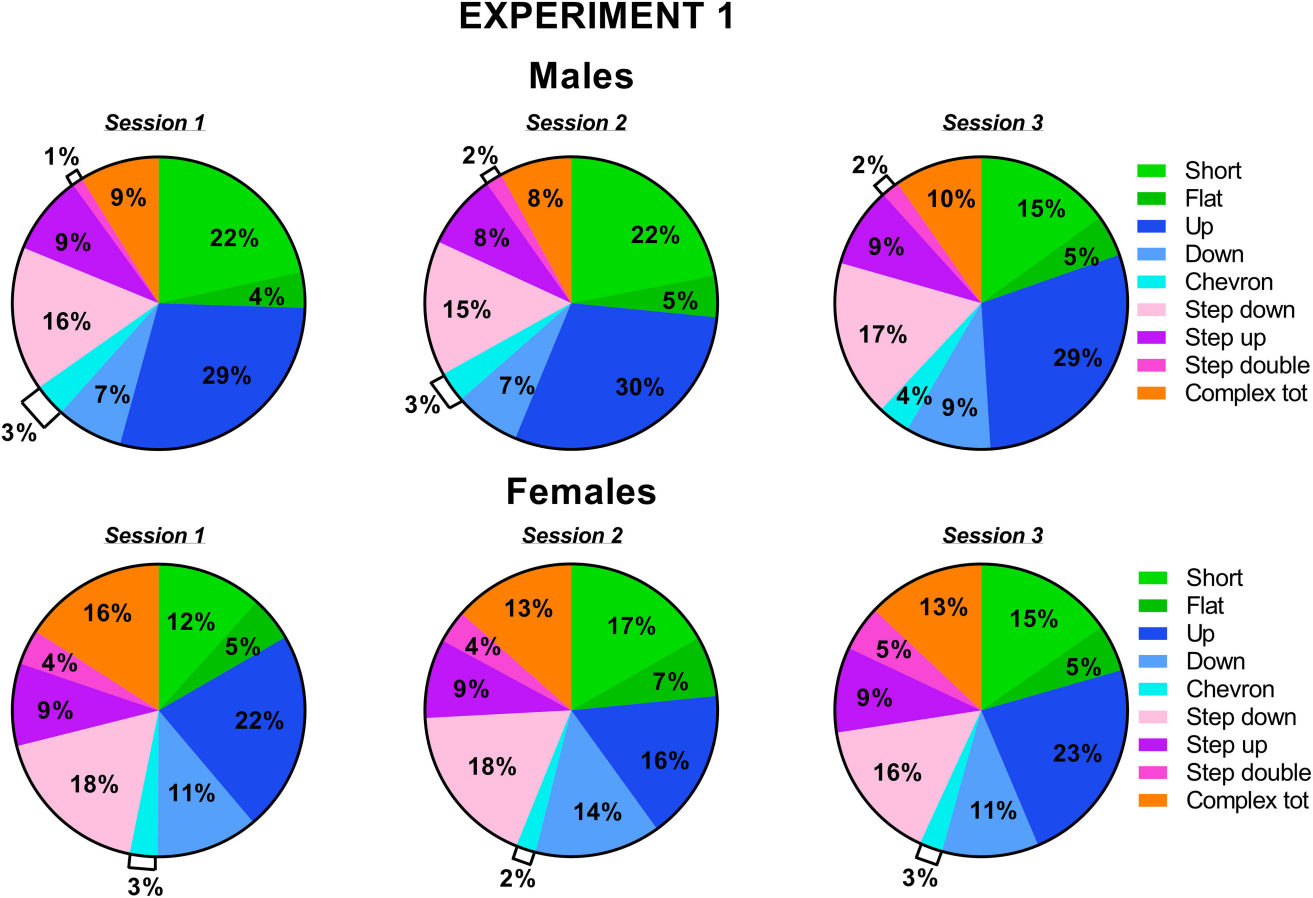
Pie charts depicting sex differences in ultrasonic call types in experiment 1 (same pre-testing isolation). Distribution of call categories in each sex and testing session. Data are expressed as percentages over the total number of USVs for each sex and session. Data are mean ± SEM. *N* = 10 for each sex. Complex tot = complex 3 + complex 4 + complex 5 (refer also to Figure 1).

The types of ultrasonic calls, as classified based on most common spectrographic categories, differed between sexes, and this pattern of results seemed more evident on the first 2 testing sessions (Figures 3, 4). The female mice tended to emit less “short” calls on the first testing session than the male mice [interaction sex × session: *F*_(2, 36)_ = 2.73, *p* = 0.08, sex effect on session 1: *F*_(1, 18)_ = 3.17, *p* = 0.09; Figure 3A]. Especially during the second session, the female mice also produced overall less “up” calls [sex effect: *F*_(1, 18)_ = 7.27, *p* < 0.05; interaction sex × session: *F*_(2, 36)_ = 3.05, *p* = 0.06, sex effect on session 2: *F*_(1, 18)_ = 16.01, *p* < 0.001; Figure 3C] and more “down” calls [sex effect: *F*_(1, 18)_ = 4.91, *p* < 0.05; interaction sex × session: *F*_(2, 36)_ = 3.2, *p* = 0.05, sex effect on session 2: *F*_(1, 18)_ = 10.93, *p* < 0.01; Figure 3D]. The female mice also emitted more “step double and more “complex” calls with 3 or more components [sex effect, respectively: *F*_(1, 18)_ = 11.44 and 7.3, *p* < 0.05; Figures 3H,I].

#### The Effects of Estrous Phase: Social Interaction and USVs in Female Mice

While the male B6 mice were all tested with a female CD1 stimulus in diestrous phase, the female B6 mice were tested on each session with a female CD1 either in estrous or diestrous phase, with a balanced assignment across sessions (refer also to Table 1). The estrous phase of the stimulus females did not affect the social behaviors in any of the testing sessions, neither any of the USVs' characteristics (stimulus' estrous phase effect for each testing session: all ns).

**Figure 5:**
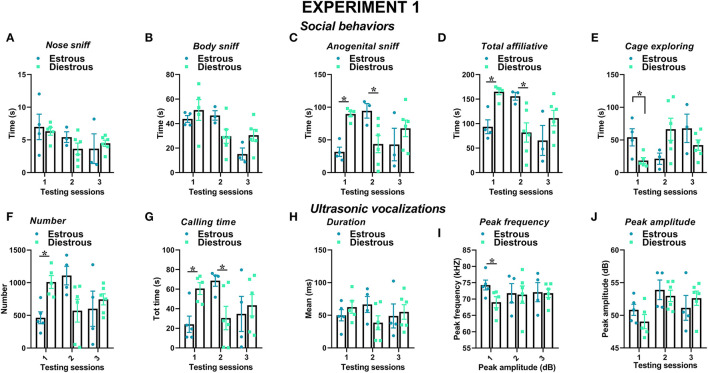
Effects of the estrous phase of the resident female mice on social behaviors and USVs in experiment 1 (same pre-testing isolation). The impact of the estrous phase of the experimental female B6 subjects was investigated on each testing day on social behaviors **(A–E)** and USVs' characteristics **(F–J)**. All the B6 mice were isolated for 3 days in the testing cage before each social encounter. For the exact number of mice in each estrous phase, refer to Table 1. Data are mean ± SEM. Total *N* = 10 before exclusion of statistical outliers by Grubb's test for small samples.*p < 0.05.

The estrous phase of the experimental B6 female mice affected their social behaviors on the first 2 testing sessions (Figures 5A–E): the female mice in estrous phase displayed less anogenital sniffing on session 1 and more on session 2 than those in diestrous [estrous phase effect on sessions 1 and 2, respectively: *F*_(1, 7)_ = 55.3 and 5.96, *p* < 0.001 and < 0.05; Figure 5C], and the same pattern was observed for total affiliative behaviors [estrous phase effect on sessions 1 and 2, respectively: *F*_(1, 7)_ = 24.97 and 6.39, *p* < 0.01 and < 0.05; Figure 5D]. In parallel, cage exploration was more evident in the estrous than in the diestrous female mice in session 1 [estrous phase effect on session 1: *F*_(1, 7)_ = 7.89, *p* < 0.05; Figure 5E] and tended to decrease afterward.

The estrous phase of the experimental B6 female mice also affected certain characteristics of the USVs they emitted, especially in the first testing session (Figures 5F–J). The female mice in estrous phase emitted less USVs in session 1 than those in diestrous phase [estrous phase effect on session 1: *F*_(1, 8)_ = 16.74, *p* < 0.05; Figure 5F]. The female mice in estrous phase also spent less time calling in session 1 compared to those in diestrous phase, while an increase was observed in session 2 [estrous phase effect on s1 and s2, respectively: *F*_(1, 8)_ = 12.50 and 5.99 *p* < 0.05; Figure 5G]. The peak frequency of the USVs emitted by the female mice in estrous phase was also higher, although this effect was again detectable only in session 1 [estrous phase effect on session 1: *F*_(1, 8)_ = 5.35, *p* < 0.05; Figure 5I]. No difference was found in the distribution of call types between estrous and diestrous experimental female subjects (Supplementary Figure 2).

### Experiment 2: Different Pre-Testing Isolation Time in Male and Female Mice

#### Sex Differences: Social Interaction and USVs

**Figure 6:**
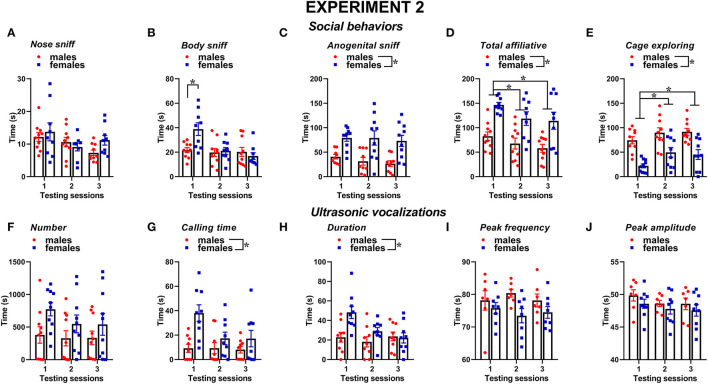
Sex differences in social behaviors and USVs in experiment 2 (different pre-testing isolation). **(A–E)** Social behaviors and **(F–J)** USVs of the male and female experimental B6 subjects were assessed by analysis of the video recordings (Observer, Noldus) and spectrograms (Avisoft SASLab Pro), respectively. The B6 female mice were isolated for 3 days in the testing cage before each social encounter as in experiment 1, while the male mice were isolated for only 10 min pre-testing, as in most commonly used procedures. As in experiment 1, social behavior and USVs were analyzed during 3 testing sessions of 3 min each (inter-session interval: 7–10 days) using an adult female CD1 as stimulus. Data are mean ± SEM. *N* = 10 before exclusion of statistical outliers by Grubb's test for small samples.*p < 0.05. Sex differences are reported as * in each graph legend when a significant main effect of sex was detected in the absence of any interaction with testing session.

Similar to experiment 1, also, when the male mice were not isolated before testing, social behaviors were overall more expressed by the female mice than the male one and tended to decrease with testing sessions; furthermore, sex differences depended on specific type of behavior (Figures 6A–E). The female mice displayed more body sniffing than the male mice, especially in the first testing session [interaction sex × session: *F*_(2, 34)_ = 5.34, *p* < 0.01; sex effect on session 1: *F*_(1, 17)_ = 10.36, *p* < 0.01; Figure 6B]. The female mice were also overall engaged in more anogenital sniffing than the male mice [sex effect: *F*_(1, 17)_ = 16.72, *p* < 0.01, Figure 6C], an effect that was stable across the sessions. The female mice display more affiliative behaviors and explored significantly less the testing cage than the male mice [sex effect, respectively: *F*_(1, 17)_ = 9.61 and *F*_(1, 18)_ = 20.51, *p* < 0.01; Figures 6D,E]. In mice of both sexes, the levels of affiliative behaviors tended to decrease with testing sessions, while those of cage exploration increased [session effect, respectively: *F*_(2, 34)_ = 6.55 and *F*_(2, 36)_ = 10.98, *p* < 0.01; Figures 6D,E].

Several characteristics of USVs differed between the two sexes (Figures 6F–J), in a highly similar manner to what was observed in experiment 1. Although the number of USVs emitted was not significantly different (Figure 6F), the total calling time and the mean duration were higher in the female mice [sex effect, respectively: *F*_(1, 16)_ = 6.6 and 5.32, *p* < 0.05; Figures 6G,H]. All the USV parameters did not significantly change across the testing sessions (session effect and its interaction with sex: all ns).

**Figure 7:**
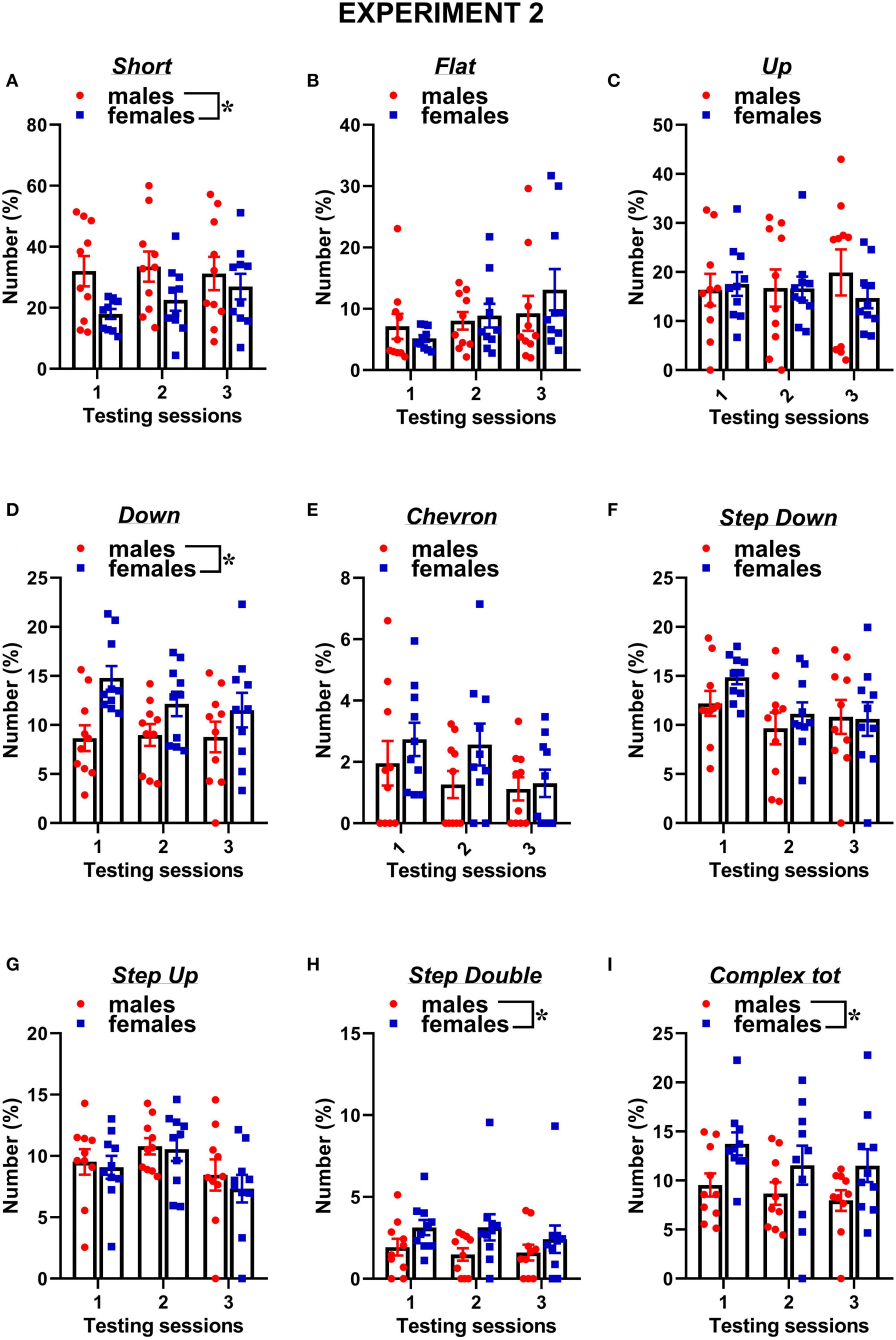
Sex differences in ultrasonic call types in experiment 2 (different pre-testing isolation). **(A-I)** The different call types were automatically classified (refer to Figure 1 for a detailed description). Complex tot = complex 3 + complex 4 + complex 5. Data are expressed as percentages over the total number of USVs for each sex and session. Data are mean ± SEM. *N* = 10 for each sex. *p < 0.05.* Refers to a nonsignificant tendency (0.05 < *p* ≤ 0.09). Sex differences are reported as * in each graph legend when a significant main effect of sex was detected in the absence of any interaction with testing session.

**Figure 8:**
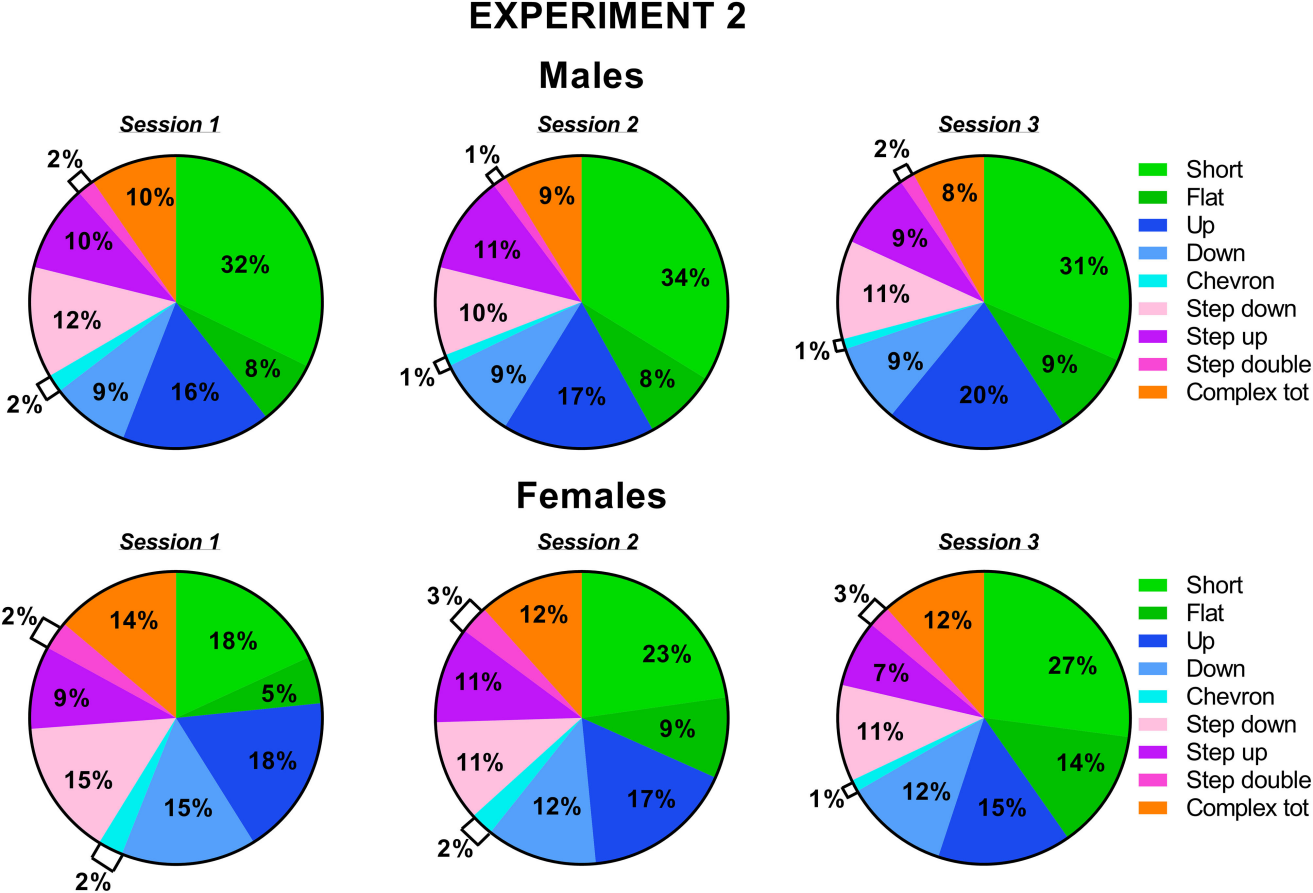
Pie charts depicting sex differences in ultrasonic call types in experiment 2 (different pre-testing isolation). Distribution of call categories in each sex and testing session. Data are expressed as percentages over the total number of USVs for each sex and session. Data are mean ± SEM. *N* = 10 for each sex. Complex tot = complex 3 + complex 4+ complex 5 (refer also to Figure 1).

The classification of the call types revealed several sex differences that were mostly independent of the testing sessions (Figures 7, 8). The female mice tended to emit less “short” calls than the male mice [sex effect: *F*_(1, 18)_ = 3.53, *p* = 0.08; Figure 7A]. The female mice also produced more “down” calls [sex effect: *F*_(1, 18)_ = 9.15, *p* < 0.01; Figure 7D], more “step double” [sex effect: *F*_(1, 18)_ = 4.12, *p* = 0.06 Figure 7H], and more complex calls [sex effect: *F*_(1, 18)_ =7.3, *p* < 0.05; Figure 7I].

#### The Effects of Estrous Phase: Social Interaction and USVs in Female Mice

As for experiment 1, the female B6 mice were tested in each session with a female CD1 either in estrous or diestrous phase, with a balanced assignment across sessions and between resident's estrous phases (Table 1). As observed in experiment 1, the estrous phase of the female stimulus (intruder) did not affect the social behaviors in any of the testing sessions or any of the USV characteristics (stimulus' estrous phase effect for each testing session: all ns).

**Figure 9:**
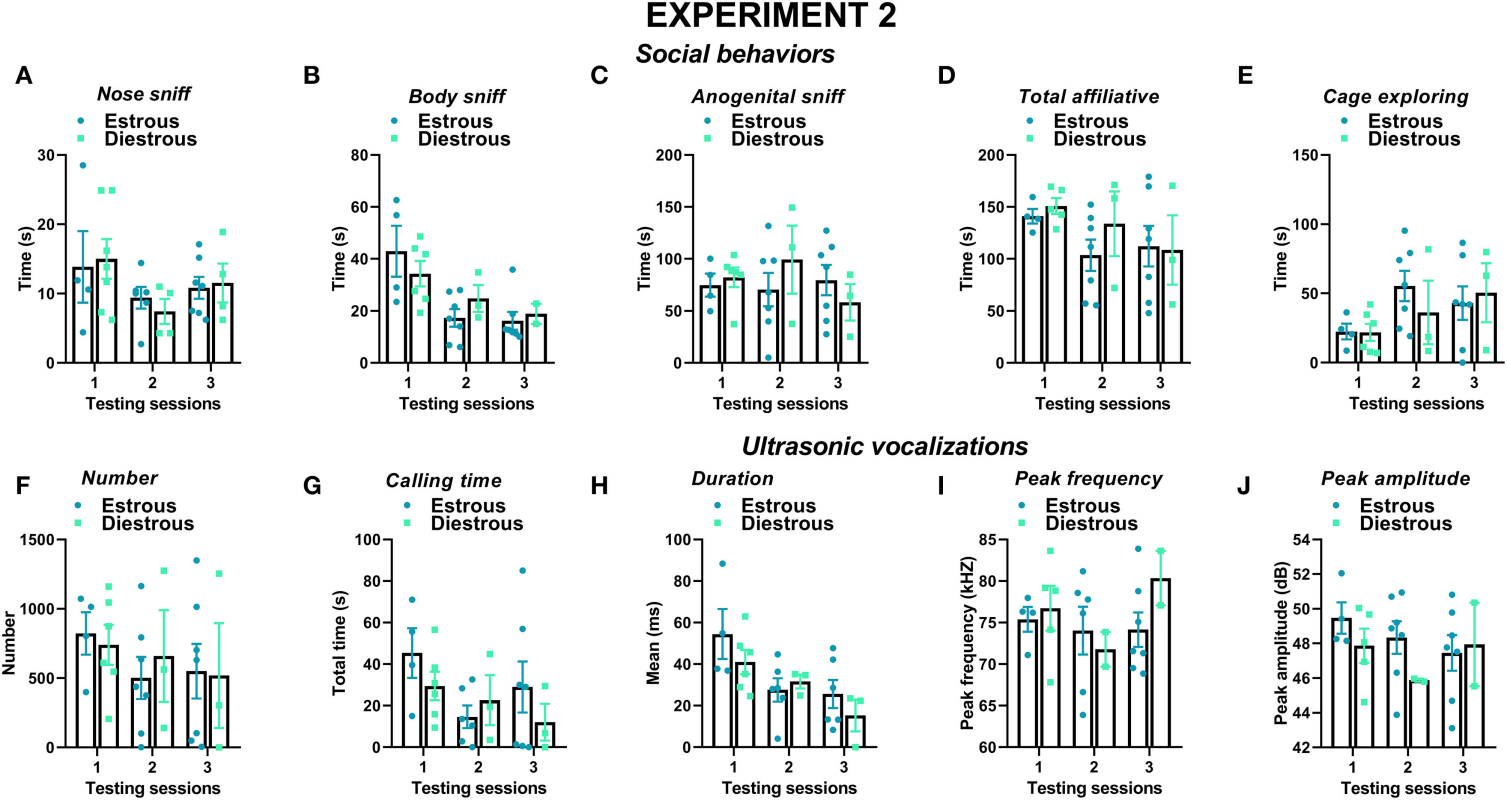
Effects of the estrous phase of the resident female mice on social behaviors and USVs in experiment 2 (different pre-testing isolation). The effects of the estrous phase of the experimental female B6 subjects were investigated on each testing day on **(A–E)** social behaviors and **(F–J)** multiple USV characteristics. The B6 female mice were isolated for 3 days in the testing cage before each social encounter, as in experiment 1, while the male mice were isolated for only 10 min pre-testing, as in most standard protocols. Social behaviors and USVs were analyzed following otherwise the same procedures used for experiment 1 (3 testing sessions of 3 min each and at an interval of 7–10 days). A detailed distribution of the estrous phase in the female residents and intruders is provided in Table 1. Data are mean ± SEM. *N* = 10 before exclusion of statistical outliers by Grubb's test for small samples.

In contrast to what was observed in experiment 1, the estrous phase of the female resident did not modulate any of its social behaviors in any testing session (effects of estrous phase on all sessions: ns; Figures 9A–E). Furthermore, no significant effect of the resident's estrous phase was found on any of the USV characteristics (effects of estrous phase on all sessions: ns; Figures 9F–J) or on their composition based on call types (Supplementary Figure 3).

### Comparison Between Experiments 1 and 2 in Female Mice: Social Interaction and USVs

The female mice in both experiments were tested under the same experimental conditions, i.e., following a 72-h isolation period before social encounters. Since the female subjects belonged to two independent cohorts, in order to evaluate the replicability of female phenotypes, we analyzed the female dataset by an additional ANOVA with experiment as the between-subject factor and testing session as the within-subject variable.

Concerning social behaviors, the results on session-dependent changes were similar between the experiments, as shown by lack of the interaction experiment × session (all effects, n.s.). Independently of the experiment, the levels of affiliative behaviors tended to decrease with the testing sessions while those of cage exploration increased [session effect, respectively: *F*_(2, 32)_ = 10.31 and *F*_(2, 34)_ = 10.47, *p* < 0.001; Figures 2, 6]. Independently of the experiments, all the USV parameters did not significantly change across the testing sessions (Figures 2, 6), and the expression of the different call types (Figures 3, 4, 7, 8; session effect and its interaction with experiment: all ns). Significant interaction experiment × session was only found in peak amplitude that tended to increase from the first to the second session but only in the first experiment [*F*_(2, 34)_ = 5.5, *p* < 0.05; Figure 2J].

The effects of the estrous phase of the resident female mice were instead statistically different between the two experiments, both on social behaviors and USVs parameters, as expected because of the presence of estrous phase effects in the first but not in the second experiment. A significant interaction experiment × estrous phase was found in the time spent in anogenital sniffing, affiliative behaviors, and cage exploration (Figures 5, 9) in session 1 [*F*_(1, 15)_ = 8.62, 9.28, 5.27; *p* < 0.05] and in affiliative time in session 2 [*F*_(1, 15)_ = 6.14; *p* < 0.05]. Concerning the USV parameters (Figures 5, 9), a significant interaction experiment × estrous phase was found for session 1 in the number of USVs and calling time [*F*_(1, 16)_ = 6.13, 10.2; *p* < 0.05].

## Discussion

Our findings provide convincing evidence for sex differences in ultrasonic communication and social interaction in the C57BL/6J mouse strain. In the context of male-female vs. female-female interactions, differences in ultrasonic communication between sexes were mainly qualitative, while those in social behaviors were both quantitative and qualitative. Sex differences were highly similar between the two experiments, i.e., their detection was not substantially affected by differences in pre-testing isolation of the male mice. Nonetheless, subtle differences in the social and ultrasonic profiles of the male mice emerged between the two experiments, suggesting an impact of pre-testing social isolation on the male mice. Sex differences were mostly stable across the three testing sessions with an unfamiliar intruder, although an overall tendency to social habituation occurred in both experiments and sexes.

The estrous cycle of the resident female mice altered the social behaviors and ultrasonic communication of the female mice, although these effects were significantly detected only in the first experiment. Here, both quantitative and qualitative differences were indeed observed between receptive and non-receptive female residents, while the estrous phase of the intruder did not modulate any of the considered behavioral parameters.

### Sex Differences and Isolation Effects (Comparison Between Experiments 1 and 2)

Sex differences in social behaviors and ultrasonic communication were overall highly comparable between the two experiments, with the female mice displaying more affiliative behaviors and less cage exploration than the male ones while emitting longer USVs and with less simple one-component calls (e.g., “short” and “up”) but more complex calls (e.g., “step double” and “total complex”). Nonetheless, subtle additional sex differences were found only in experiment 1, including a male-specific decrease with testing sessions in anogenital sniffing and USV number, as well as an overall higher peak frequency of male USVs. Hence, our data suggest that the experimental settings used in our experiment 1, including a resident-intruder context with 72-h pre-testing isolation, may be the most suitable to detect both major and minor sex differences in social and ultrasonic behaviors. Since the female mice were tested under exactly the same experimental conditions in experiment 2, it is natural to infer that the differences emerging between our two experiments are due to corresponding differences in the behaviors of the male mice that were exposed to pre-testing isolation only for experiment 1. The behavior of the female mice was indeed highly comparable in our two experiments, as confirmed by the statistical comparison of the two female datasets, supporting the replicability of female social and ultrasonic behavioral profiles across the repeated testing sessions. Male behaviors appeared instead slightly different between the two experiments, although we could not conduct a statistical quantification of these differences because of the confounding effects of independent testing on social isolation.

Nonetheless, the visual comparison of Figure 2 with Figure 6 and Figure 3 with Figure 7 clearly shows that the male mice in experiment 1, i.e., with longer pre-testing isolation, displayed higher levels of anogenital sniffing, more USVs, and more one-component ‘up” calls, suggesting a higher expression of these behaviors in territorial, i.e., isolated, male mice. The hypothesis of a territoriality effect of social isolation is further supported by the predominance of the differences between the two experiments during the first testing session, since this effect may be attenuated by repeated experience of social encounters with an intruder. Furthermore, it should be noted that a resident-intruder setting was employed only in experiment 1, while experiment 2 used a basically neutral testing environment as a consequence of the short pre-testing isolation (10 min). The reason for this experiment design was intrinsically related to the major aim of our study, which was not to specifically investigate the effects of social isolation on ultrasonic communication in male and female mice, as previously conducted by others (e.g., Zhao et al., 2021; this also explains the lack of an additional female group with minimal pre-testing isolation in our design). Instead, our goal was to evaluate sex differences either under the exact same experimental conditions for male and female mice (i.e., in the resident-intruder paradigm) or under the experimental conditions most suitable and commonly used in research studies on USVs in ASD mouse models (i.e., in a resident-intruder setting for female mice and with a short habituation to the testing environment in the case of male subjects).

Interestingly, when the effects of 72-h social isolation were previously assessed, only subtle changes were described in male B6 mice (Zhao et al., 2021). These discrepancies may be due to the longer duration of the testing session used in the previous study (i.e., 30 vs. 3 min in ours): Zhao et al. indeed described no effects of isolation on the number of USVs emitted by a male mouse toward a female intruder when the entire session was considered, but they detected a significant increase in isolated vs. grouped male mice when only the first 5 min of the session was analyzed, and they described a higher first latency to USV emission in the isolated male mice (with an average value of ~3 min, i.e., the duration of our testing session). Differences in the estrous cycle of the female intruder could also contribute to the discrepant outcomes of ours and Zhao's study on social non-vocal behaviors of isolated male mice: the authors reported a tendency, although not significant (*p* = 0.08) to an overall increase in the time spent in social interaction in isolated male mice compared to grouped ones that was accompanied by an increase in the occurrence of mounting behavior. The higher engagement in mounting of their isolated male mice could have attenuated the isolation effects on affiliative behaviors that we instead found in our study. We did not detect mounting in our tested male mice, and this is not surprising considering our short testing duration and the non-receptive estrous state of our female intruders (the estrous cycle was not assessed in Zhao's study). In conclusion, our results from the male mice and their comparison with previous findings suggest that 3 days of isolation of the male mice increases social affiliation and promotes USV emission during the initial phases of the social encounter with a female mouse. Nonetheless, the duration of the testing session and the estrous cycle of the intruders may critically influence the social effects of isolation, an issue that deserves to be specifically investigated in future studies.

Independently of the experiments, our findings suggest that the major sex differences affecting mouse ultrasonic communication are of qualitative nature (duration and call composition) rather than quantitative. While the presence of longer USVS in the female mice was in line with previous studies (e.g., von Merten et al., 2014), the lack of sex difference in the number of USVs that we found here is in disagreement with a previous study reporting that female mice emitted more USVs than male mice toward a female intruder (Hammerschmidt et al., 2012). This discrepancy may be due to the different substrain used in this study, since B6/N and B6/J are known to have markedly different ultrasonic profiles. Indeed, in B6/J mice, another study described no difference in the number of USVs (Matsumoto and Okanoya, 2018) but a reduced number of short calls and prevalence of complex calls in female mice. As short calls, together with simple calls in general, have been detected especially under territorial conditions, e.g., in male-male interactions (Matsumoto and Okanoya, 2018) and following long-term male isolation (Chabout et al., 2012), it is possible that male mice preferentially communicate using short calls. In contrast, complex calling bouts may be useful in maintaining the group structure necessary for female mice and promote interactions and cooperation (Matsumoto and Okanoya, 2018), in agreement with previous studies showing that this type of call is more attractive for female mice (Chabout et al., 2015). It is indeed increasingly accepted that ultrasonic calls from female mice facilitate proximity between animals in order to help residents to acquire relevant social information on intruders and promote group relationships (Moles et al., 2007).

Since the strain of the mice involved in the social encounter may play a role in their ultrasonic profile and potential related sex differences, it is important to underscore that our study employed different strains for the test subjects (B6) and the stimulus mice (CD1). Although mouse USVs are often analyzed during interactions within the same strain, our experimental setting is not unusual, as it has been employed in previous studies assessing the emission of USVs by resident female mice (Maggio and Whitney, 1985) and male mice (Sugimoto et al., 2011) during dyadic interactions. One study in particular (Sugimoto et al., 2011) demonstrated a lack of USVs emitted by the female stimulus (derived from the CD1 strain) also when the “devocalized” male was of a different background (B6), i.e., under conditions highly similar to ours. Furthermore, using stimuli of a different strain to induce emission of USVs by tested subjects is a typical procedure of several studies on urine-elicited USVs (Nyby et al., 1979, 1983; Nyby, 2010). The choice of the CD1 strain as stimulus enhances the applicability of our present data to the research field of neurodevelopmental disorders. Indeed, several studies with mouse models of Autism and Fragile X syndrome obtained from the B6 background have performed social and ultrasonic testing (in both male and female mice) through interactions with female CD1 stimuli (Hebert et al., 2014; Pietropaolo et al., 2014; Oddi et al., 2015; Gaudissard et al., 2017; Gauducheau et al., 2017; Lemaire-Mayo et al., 2017; Fyke et al., 2021).

### Estrous Cycle Effects on Social Behaviors and USVs (Experiments 1 and 2)

In both experiments, the estrous cycle of the intruders did not alter either the social behaviors or any parameter of ultrasonic communication. The estrous cycle of the residents instead modulated both behavioral domains, and these effects were more marked in experiment 1 (Figure 5) the female mice in estrous phase exhibited less affiliative behaviors in session 1 than those in diestrous phase, but this tendency inverted its direction in session 2 to return to the initial situation in session 3. These effects were mainly due to differences in anogenital sniffing. The effects of estrous cycle on social behavior followed those observed on USVs, since the number of USVs was also initially and finally lower in the estrous than in the diestrous female mice with a shift in session 2. USVs seemed more affected by the estrous cycle in session 1 when their number, call time, and peak frequency were all different between estrous and diestrous residents. The overall reduced social investigation and number of USVs of the estrous female mice are in agreement with previous reports (Moles et al., 2007) and fits with the reduced social interest of receptive female mice in a conspecific of the same sex. It has been suggested that oxytocin mediation of social processes is likely to play a role in the effects of the estrous phase, since it is known to be regulated by ovarian circulation (Choleris et al., 2003). Nonetheless, we failed to replicate the significant effects of the estrous cycle in experiment 2 despite the experimental testing conditions of the female mice being unchanged. It should be noted that the pattern of results of experiment 2 was still in line with what observed in experiment 1 (Figure 9), although the composition of the estrous vs. diestrous female in each session was less balanced than in experiment 1 (see Table 1). It is possible that the lower number of female mice, especially in the diestrous phase (3 in some testing sessions), may have limited the emergence of significant differences. In none of the experiments any effect of the estrous cycle was detected on the call types (Supplementary Figures 2, 3).

### Effects of Testing Experience at the Group and Individual Levels (Experiments 1 and 2)

We observed several group differences in our study, both in social non-vocal and vocal behaviors. It is intriguing to question whether the group differences could be confirmed at the individual level; to this end, the visual evaluation of individual plots (Supplementary Figures 4, 5) supports interesting considerations. Concerning social non-vocal behaviors, most of the female individuals showed the expected reduction with testing sessions in the time spent performing body sniffing in both experiments (Supplementary Figures 4B, 5B), while a decrease in anogenital sniffing was confirmed in the male mice only in experiment 1 (Supplementary Figure 4C). In mice of both sexes in both experiments, the levels of affiliative behaviors tended to decrease with testing sessions while those of cage exploration increased (Supplementary Figures 4D,E, 5D,E).

Concerning individual trends in USV-related parameters, in experiment 1, we confirmed that all the USV parameters did not significantly change across the testing sessions, with the exception of peak amplitude that increased from the first to the second session in mice of both sexes (Supplementary Figure 4J) and the number of USVs that decreased across the testing sessions in the male mice only (Supplementary Figure 4F). None of the session differences appeared at the individual level in experiment 2 (Supplementary Figures 5F–J), when the male mice were not isolated before testing (confirming the lack of differences observed at the group level).

Concerning the types of ultrasonic calls, it is important to underscore that the stability of call compositions in each sex across the testing sessions was confirmed at the individual level (Supplementary Figures 6, 7) when we selected the individuals with the highest total calling rates (half of each sex for each experiment). The overall higher proportion of short calls in males mice and the lower proportion of complex calls (“step double” and “complex tot”) were also evident in this subset of individuals.

## Conclusions

Our findings provide novel evidence for marked sex differences in ultrasonic communication that are mirrored by differences in other social behaviors of adult B6 mice. The replication of the sex differences with and without pre-testing isolation in the male mice suggests their strong consistency and should be taken into account for designing future studies using male and female adult mice. This could be especially important for studies on genetic mouse models of ASD or other pathologies involving communication deficits where pre-testing isolation may induce undesirable confounding effects more marked than in their wild-type littermates (Pietropaolo et al., 2008). Furthermore, our results demonstrate that the sex differences observed in ultrasonic communication and social behaviors are not limited to the first testing session and represent a stable trait that seems independent of the novelty of the social stimulus. Finally, these data clearly show the importance of an extensive qualitative analysis of ultrasonic communication in adult mice, since this approach contributes to unravel the more complex structure of female vs. male calls.

## Data Availability Statement

The raw data supporting the conclusions of this article will be made available by the authors, without undue reservation.

## Ethics Statement

The animal study was reviewed and approved by Comité d'Ethique pour l'experimentation animale de Bordeaux (CE 50) and the French Ministry (Ministere de l'enseignement superieur de la recherché et de l'innovation).

## Author Contributions

MP conducted the experiments and behavioral analyses and participated in the writing of the manuscript. VP participated in the analysis of USVs, prepared all the figures, and contributed to the writing of the results. RB classified the call types. SP designed the experiments, provided technical support for all the experiments, and wrote the manuscript. SB contributed to manuscript writing. All authors reviewed and approved the manuscript.

## Funding

SP received funding from Bordeaux University, CNRS, Association Autour de Williams and Fondation pour l'Audition (FPA-RD-2020-8).

## Conflict of Interest

RB is an employee of Metris B.V. and co-developer of the SONOTRACK Call Classification software. The remaining authors declare that the research was conducted in the absence of any commercial or financial relationships that could be construed as a potential conflict of interest.

## Publisher's Note

All claims expressed in this article are solely those of the authors and do not necessarily represent those of their affiliated organizations, or those of the publisher, the editors and the reviewers. Any product that may be evaluated in this article, or claim that may be made by its manufacturer, is not guaranteed or endorsed by the publisher.
