# Supplementary material for: Ultrasonic Vocalizations in Adult C57BL/6J Mice: The Role of Sex Differences and Repeated Testing
Source: Front Behav Neurosci. 2022 Jul 14;16:883353. doi: 10.3389/fnbeh.2022.883353 (PMC9330122; doi:10.3389/fnbeh.2022.883353)
Supplement: Supplementary file 1 [file Presentation_1.pdf]

SUPPLEMENTARY FIGURES

Ultrasonic vocalizations in adult C57Bl/6J mice: the role of sex differences and repeated testing

Marika Premoli[1], Valeria Petroni[2], Ronald Bulthuis[3], Sara Anna Bonini[1] and Susanna Pietropaolo[2]

[1]University of Brescia, Department of Molecular and Translational Medicine, Brescia, Italy

[2]Univ. Bordeaux, CNRS, INCIA, UMR 5287, F-33000 Bordeaux, France

[3]Sonotrack, Metris B.V., The Netherlands

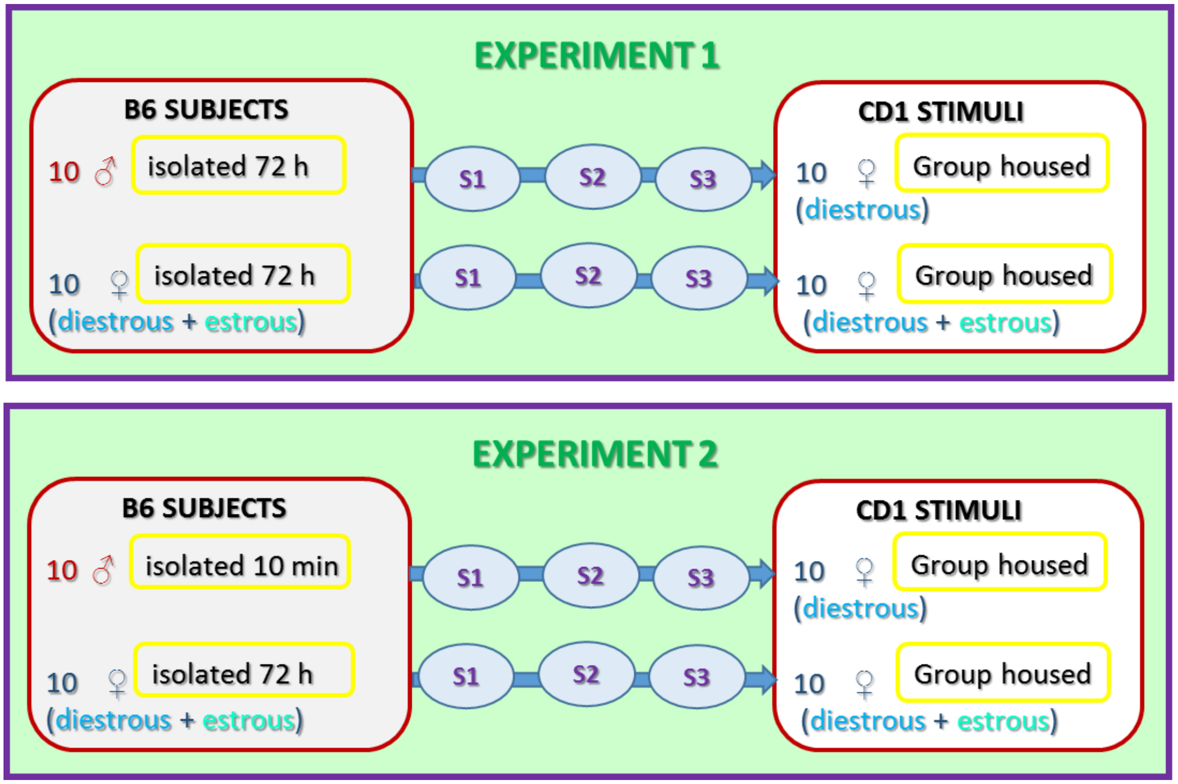

Fig. S1. Schematic representation of the experimental procedures. B6 female and male mice encountered CD1 stimulus females during 3 testing sessions of 3 min each (S1-S3, at 7-10 days of interval). Experiment 1 investigated sex differences in social behaviors and USVs in the same resident-intruder conditions, i.e., after 72 h isolation in the testing cage. Experiment 2 intended to assess sex differences using procedures commonly used to assess USVs in mouse models of ASD, i.e., in resident-intruder settings for females and with minimal isolation in the testing cage for males (i.e., 10 min habituation). During inter-session intervals all mice were grouped housed with their original groupmates. Novel CD1 stimulus females were used for each encounter to avoid social habituation. CD1 females were all naïve to social experience with B6 mice at the time of the first testing session. Each stimulus female was employed for a total of 3 times for each experiment, but only once for each testing session. Separate batches of CD1 females were employed for male-female and female-female interactions in each experiment.

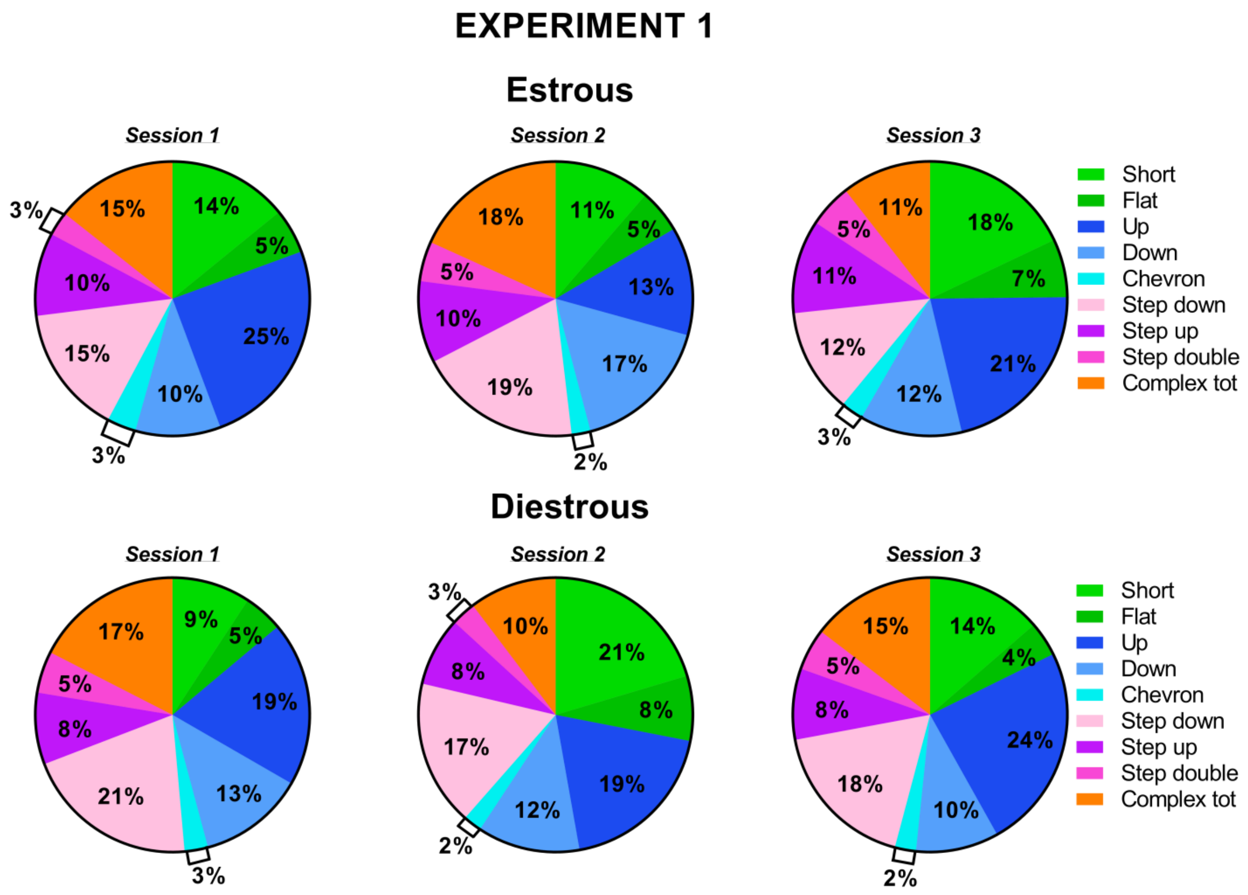

Fig. S2. The effects of the estrous phase of the resident female on ultrasonic call types in experiment 1 (same pre-testing isolation). B6 female and male mice were isolated during 3 days in the testing cage before each social encounter with a stimulus CD1 intruder. The effects of the estrous phase of the experimental female B6 subject were investigated on each testing day on the qualitative composition of USVs (described in Fig.1). Complex tot=complex3+ complex4+complex5 (see also Fig.1). The distribution of call categories for each testing session and estrous phase is illustrated through pie-charts. Data are expressed as mean percentages over the total number of USVs for each sex and estrous phase. A total of 10 females were included in the dataset; their distribution across estrous phases is detailed in Table 1.

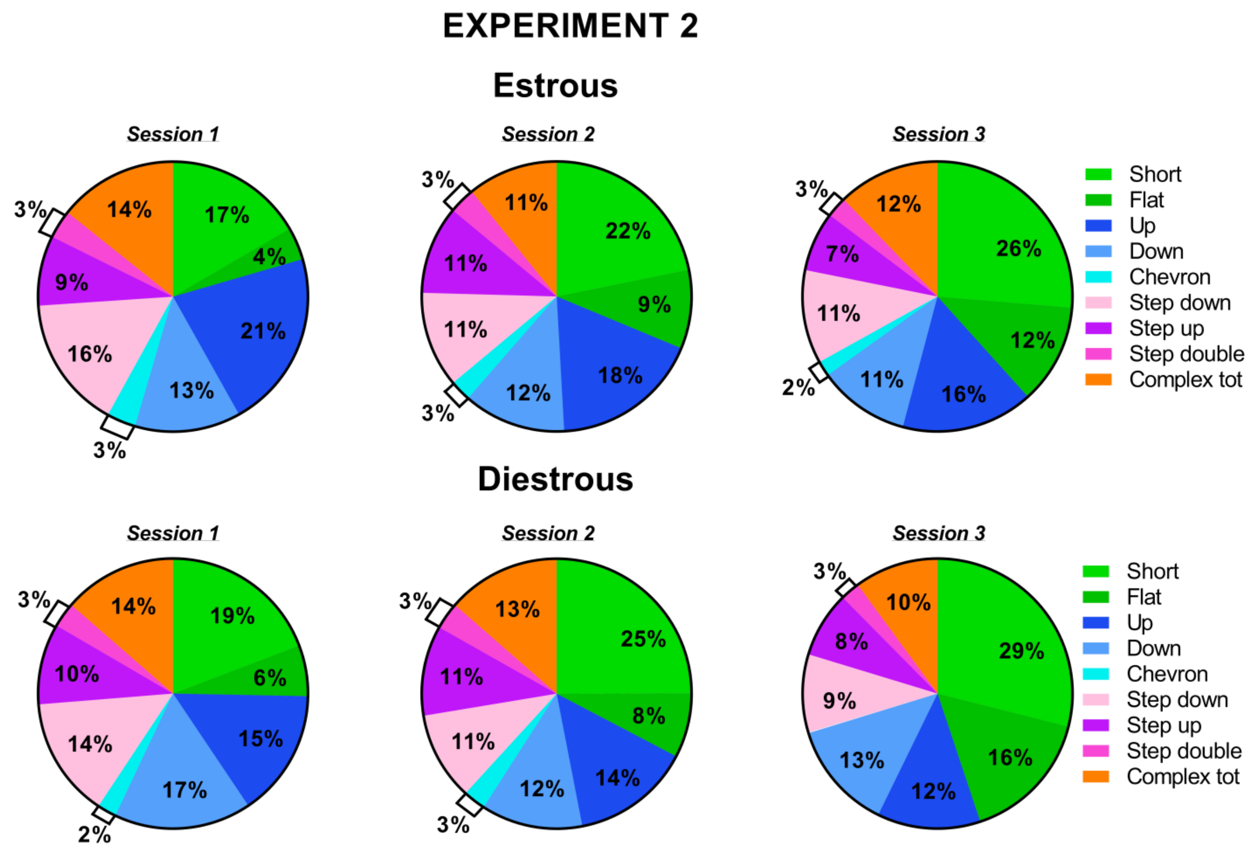

Fig. S3. The effects of the estrous phase of the resident female on social behaviors and USVs in experiment 2 (different pre-testing isolation). B6 female mice were isolated during 3 days in the testing cage before each social encounter as in experiment 1, while males were isolated only 10 min pre-testing, as in most commonly used experimental protocols. USVs were analyzed through 3 testing sessions of 3 min each, at an interval of 7-10 days. Pie-charts illustrate the percentages of each call type calculated over the total number of USVs for each estrous phase and session (mean values). A total of 10 female B6 resident subjects was included in the dataset (for the precise n of each estrous phase, see Table 1). Complex tot=complex3+ complex4+complex5 (see also Fig.1).

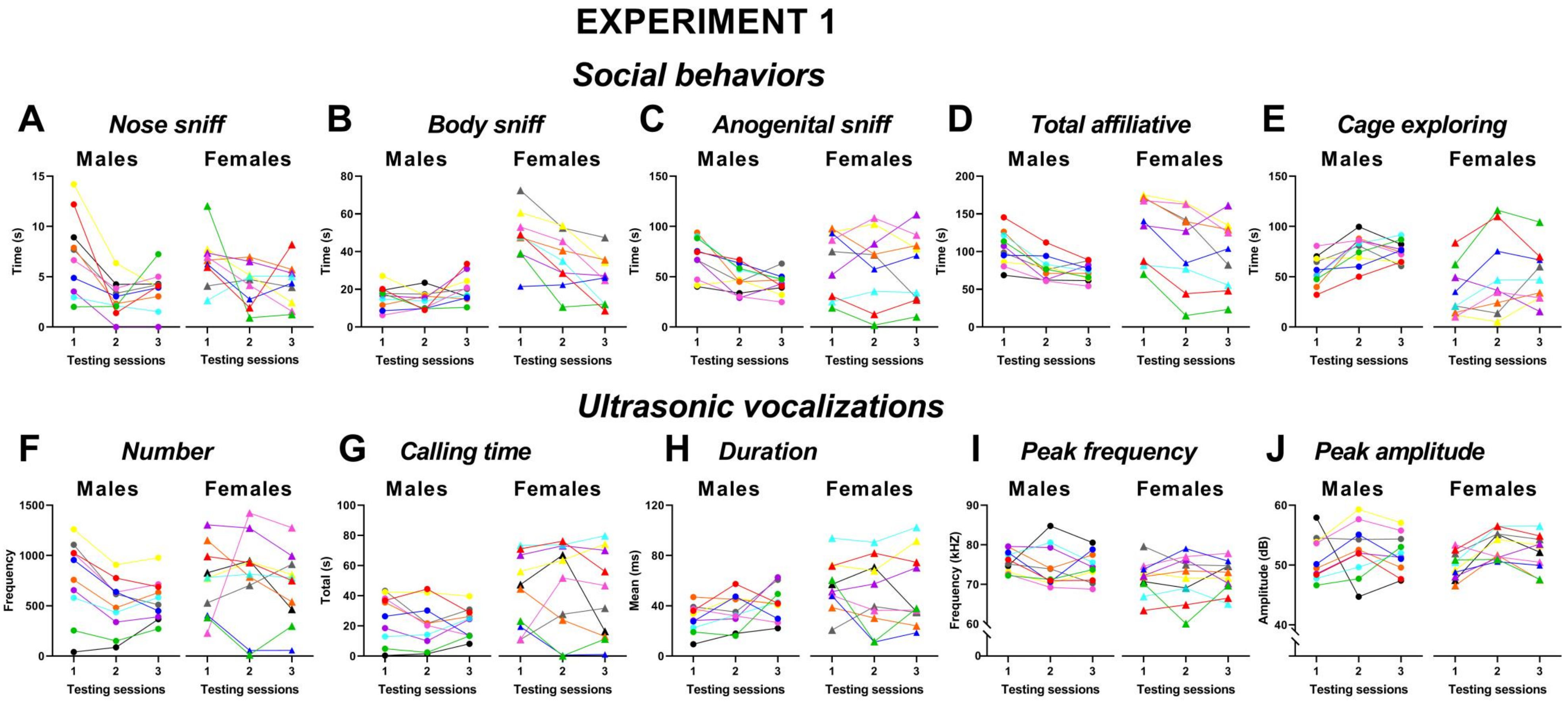

Fig. S4. Individual line plots of social behaviors and ultrasonic communication in experiment 1 (same pre-testing isolation).

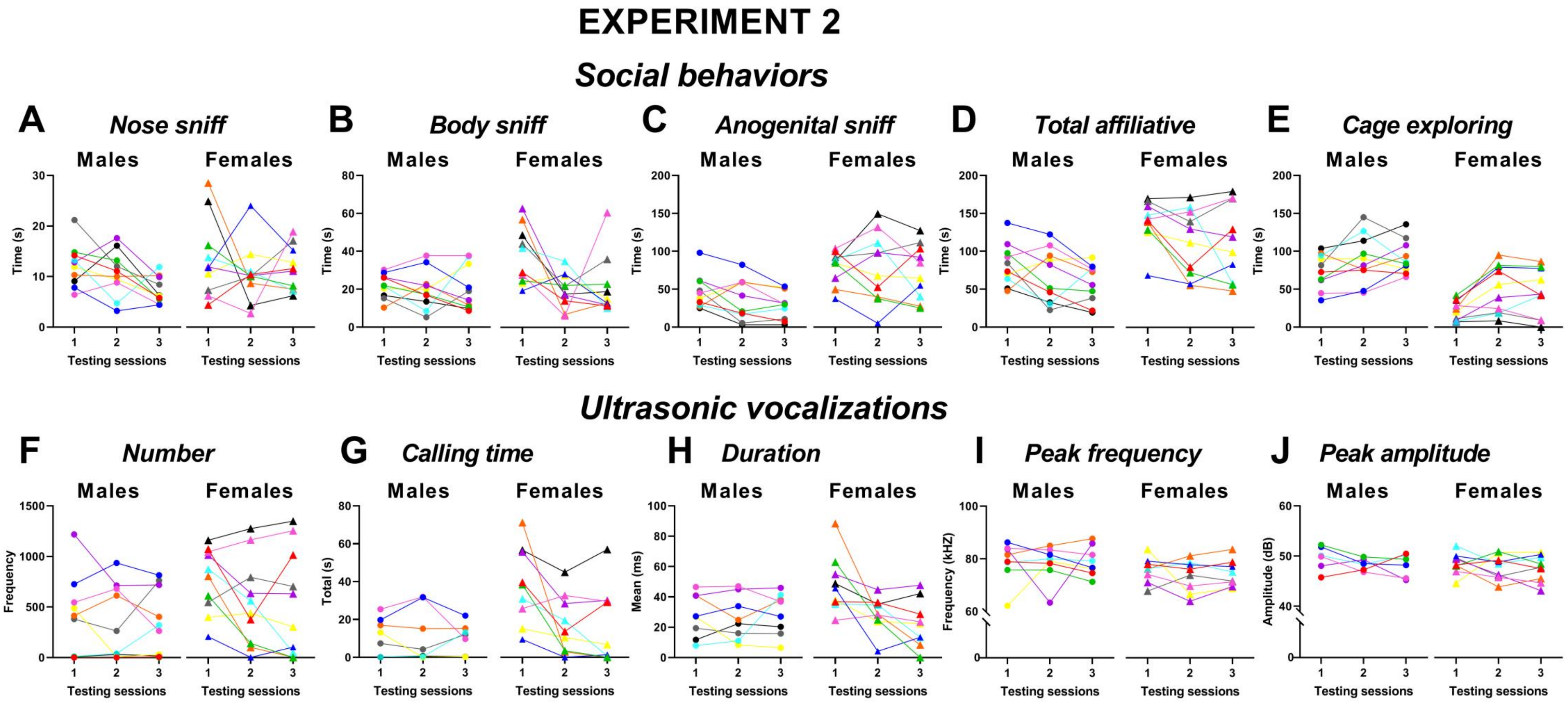

Fig. S5. Individual line plots of social behaviors and ultrasonic communication in experiment 2 (different pre-testing isolation).

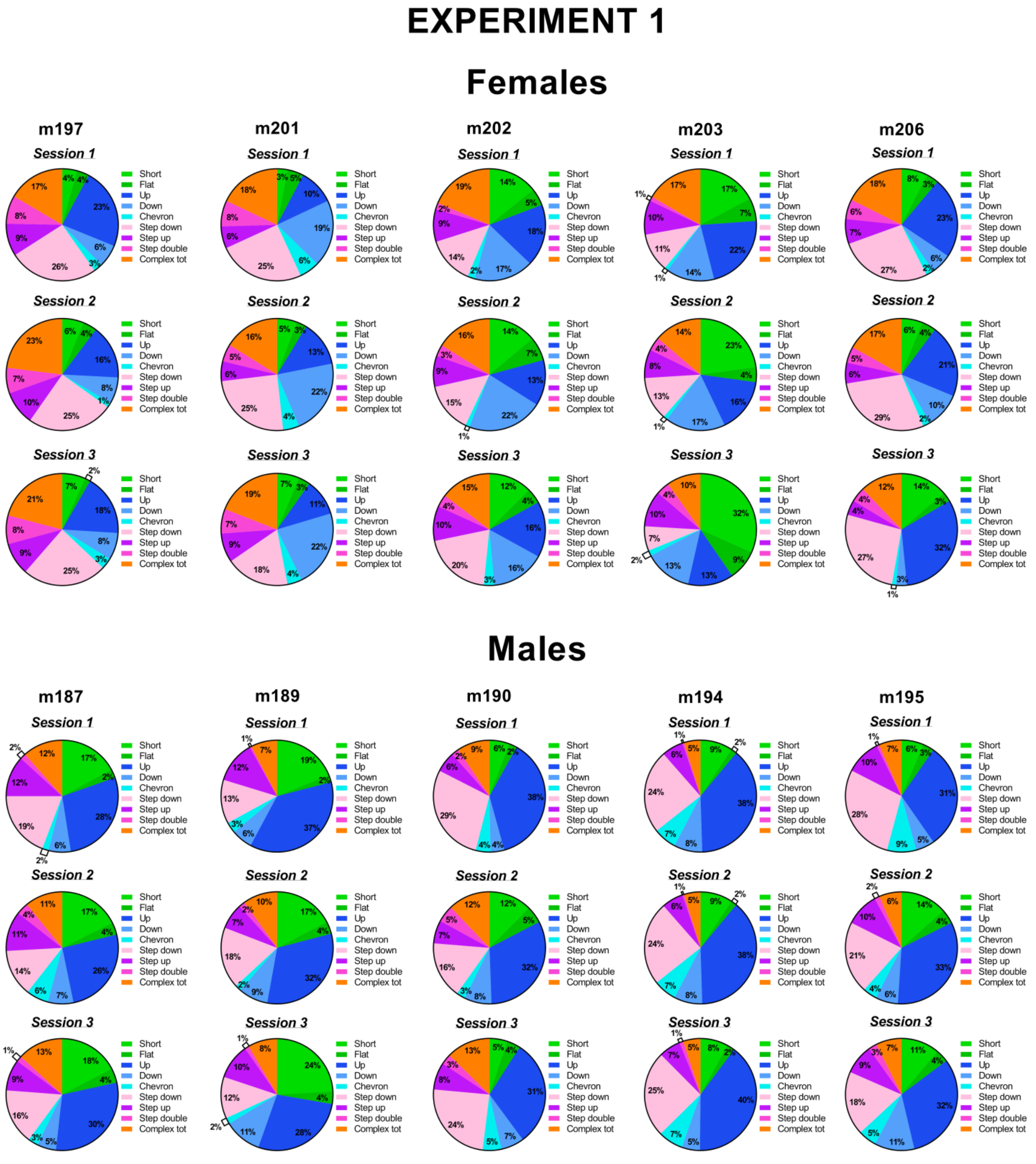

Fig. S6. Stability of call type composition across testing sessions in high emitting individuals from experiment 1 (same pre-testing isolation).

In order to evaluate whether the stability of call composition across testing sessions was confirmed at the individual level, we selected 5 individuals for each sex with the highest total number of USVs. Pie-charts illustrate the percentages of each call type calculated over the total number of USVs for each testing session. M= mouse.

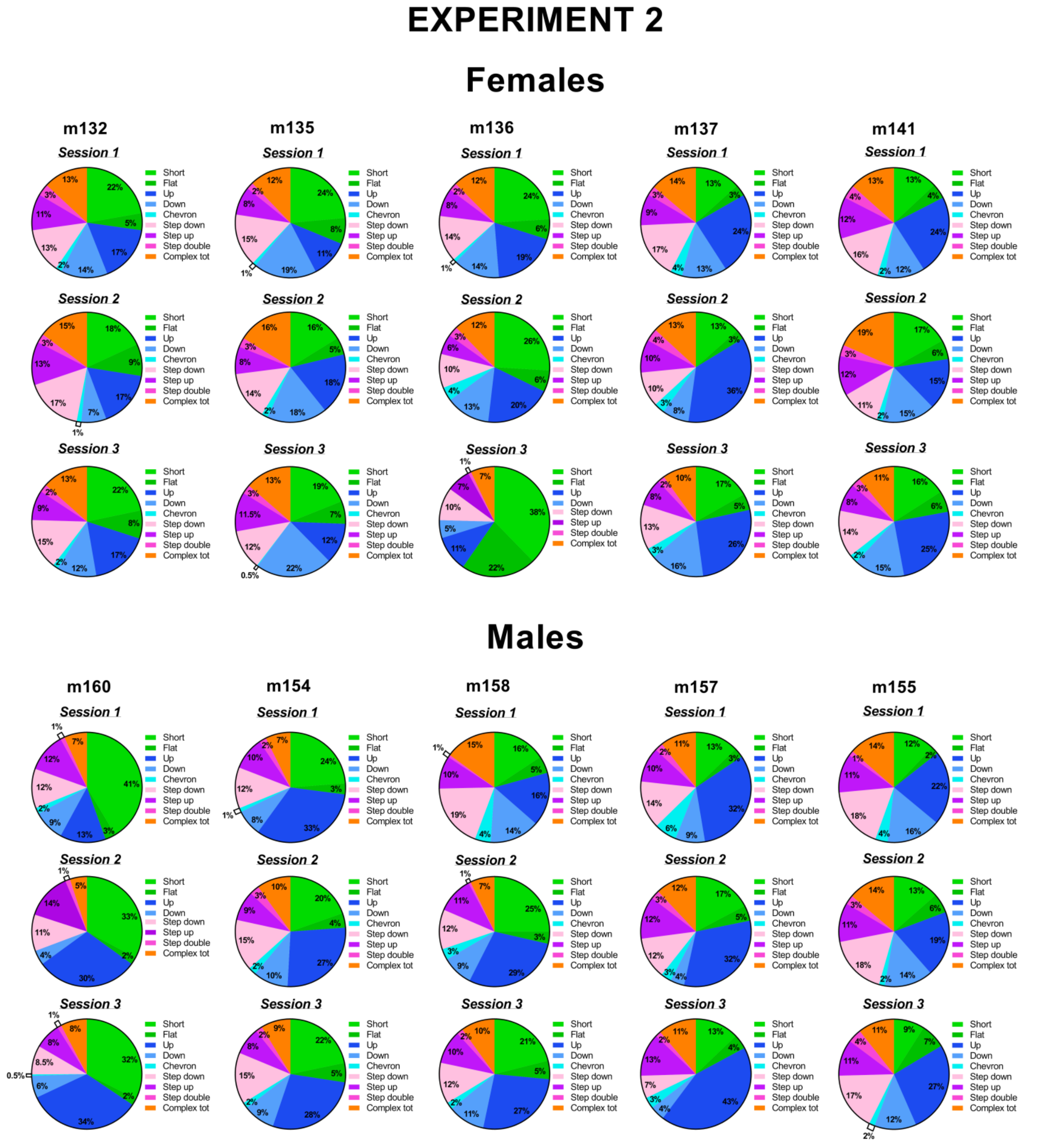

Fig. S7. Stability of call type composition across testing sessions in high emitting individuals from experiment 2 (different pre-testing isolation). Individual patterns of changes in call composition across testing sessions were evaluated in 5 individuals for each sex emitting the highest total number of USVs. Pie-charts illustrate the percentages of each call type calculated over the total number of USVs for each session. M= mouse.
